# Measuring Greenspace in Rural Areas for Studies of Birth Outcomes: A Comparison of Street View Data and Satellite Data

**DOI:** 10.1029/2024GH001012

**Published:** 2024-03-28

**Authors:** Xun Shi, Fan Zhang, Jonathan W. Chipman, Meifang Li, Camilo Khatchikian, Margaret R. Karagas

**Affiliations:** ^1^ Department of Geography Dartmouth College Hanover NH USA; ^2^ School of Earth and Space Sciences Institute of Remote Sensing and Geographical Information System Peking University Beijing China; ^3^ Department of Epidemiology Geisel School of Medicine at Dartmouth Lebanon NH USA; ^4^ Children’s Environmental Health and Disease Prevention Research Center at Dartmouth Hanover NH USA

**Keywords:** greenspace, street view, diversity, birth outcomes, children’s health, rural

## Abstract

Using street view data, in replace of remotely sensed (RS) data, to study the health impact of greenspace has become popular. However, direct comparisons of these two methods of measuring greenspace are still limited, and their findings are inconsistent. On the other hand, almost all studies of greenspace focus on urban areas. The effectiveness of greenspace in rural areas remains to be investigated. In this study, we compared measures of greenspace based on the Google Street View data with those based on RS data by calculating the correlation between the two and evaluating their associations with birth outcomes. Besides the direct measures of greenness, we also compared the measures of environmental diversity, calculated with the two types of data. Our study area consists of the States of New Hampshire and Vermont, USA, which are largely rural. Our results show that the correlations between the two types of greenness measures were weak to moderate, and the greenness at an eye‐level view largely reflects the immediate surroundings. Neither the street view data‐ nor the RS data‐based measures identify the influence of greenspace on birth outcomes in our rural study area. Interestingly, the environmental diversity was largely negatively associated with birth outcomes, particularly gestational age. Our study revealed that in rural areas, the effectiveness of greenspace and environmental diversity may be considerably different from that in urban areas.

## Key Points

- We compared two methods for measuring greenspace in rural areas based on Google Street View data and remote sensing data, respectively
- The comparison was conducted by calculating the correlation between the two and their associations with birth outcomes in NH and VT, USA
- There is a considerable difference between the two types of greenspace measures, and neither significantly impacts birth outcomes

## 1 Introduction

Verheij et al. (2008) give an interesting example of how geographic perspectives can shape our views of greenspace: A French delegation from the deprived Paris suburbs visited an equally deprived neighborhood in Rotterdam (Netherlands). The delegation concluded that the Rotterdam neighborhood was far better than theirs, because the former “has open, green spaces” and “breathes.” However, the authors of the paper found that from a satellite, the French neighborhood looked much greener than the Rotterdam neighborhood. We see this true story as an example of the notion that the horizontal (eye‐level) human perception and the vertical (bird’s‐eye) perception of greenspace as imaged by a satellite can be highly incompatible (Kang et al., 2020; Wang et al., 2021). As the pathways by which greenspace affects human health are primarily attributed to positive psychological effects and friendly environmental conditions (Lachowycz & Jones, 2013), a representation of a human’s perception should be more accurate than a bird’s perceptions in evaluating the impact of greenspace on human health.

A traditional way to take a human’s perception in the study of greenspace (or more general built environment) is the foot‐based street audit. Recently, the alternative of using digital street view image data to conduct a virtual audit has become possible (Pliakas et al., 2017). Street view data are the eye‐level panoramic images assembled from photos captured by special cameras equipped on vehicles running on streets. Ever since Google launched the first publicly accessible street view program, researchers have realized its value in public health studies and practices and tested it in various applications (e.g., Kang et al., 2020; Rzotkiewicz et al., 2018 for reviews). For example, using Google Street View (GSV) data, Odgers et al. (2012) found positive neighborhood features, including street safety and the percentage of greenspace, were associated with higher prosocial behavior and healthy weight status among children in England and Wales. They concluded that the use of GSV was a reliable and cost‐effective tool for measuring both negative and positive features of local neighborhoods. In the past few years, there has been a rapid increase in the literature on using digital street view data to measure greenspaces and then relating the measurement to various health issues, for example, depression (Helbich et al., 2019), general mental health (Dai et al., 2021; Wang et al., 2021), obesity (Xiao et al., 2020), walking behavior (Lu et al., 2018), physical activity (Lu, 2019), and sleep characteristics of children (Jimenez et al., 2022). The data employed in these studies were no longer limited to GSV, but also included Microsoft Bing (Rzotkiewicz et al., 2018), Baidu Street View (Dai et al., 2021; Wu et al., 2020; Xiao et al., 2020), and Tencent Map (Wang et al., 2021).

Nevertheless, the vertical (or orthographic) view of greenspace based on remote sensing data may currently still be the most widely used method of mapping and quantifying greenspace in the literature, either directly using aerial or satellite images or employing the land use data interpreted from such imagery (Gascon et al., 2016; Helbich et al., 2019; Markevych et al., 2017; Su et al., 2019). These methods offer the advantages of low cost, extensive spatial and temporal coverages, high temporal resolution, and relatively straightforward information retrieval and analytical procedures. Such procedures may include quantifying greenspace within a fixed radius buffer (Su et al., 2019) and calculating the distance to nearby greenspace patches (Higgs et al., 2012). When using remote sensing images to quantify greenspace, raw reflectance measurements are typically transformed into spectral vegetation indices (Huete et al., 1999), such as the Normalized Difference Vegetation Index (NDVI; Deering, 1978), measured by the difference between near‐infrared and red reflectance normalized by their sum.

Several studies have directly compared street view and remote sensing data in measuring greenspaces. Helbich et al. (2021) compared NDVI‐based and street view data‐based metrics of greenness and their associations with depressive and anxiety symptoms of adults in Amsterdam, the Netherlands. They found that first, the metrics based on the two data sets were moderately correlated; second, the street view data‐based metrics were less sensitive to spatial scales and residential contexts than the NDVI‐based metrics; and third, there was no statistically significant evidence that people with less urban residential greenness had higher depression or anxiety scores than those exposed to higher levels of greenness. They suggested that although the metrics based on different data captured different aspects of greenness, those differences did not translate into different associations with mental health outcomes. Using data from Guangzhou, China, Wang et al. (2021) compared measures of greenspace based on NDVI, street view data, and self‐reporting survey, as well as their associations with mental health. In contrast to Helbich et al. (2021), they found that the measures based on different data were not correlated with each other. They also found that only the quality measure based on street view data in recreational places was positively associated with mental health. They concluded that eye‐level greenspace quality may be more strongly related to mental health. Jimenez et al. (2022) compared the greenspace measured by NDVI and street view data in Massachusetts, USA, and investigated their associations with sleep characteristics among children. They found that the correlation between the two types of metrics of greenspace varied by type of vegetation. On some general or major metrics, for example, the total greenspace and percentage of trees, the two types of metrics are moderately correlated (the correlation varies between 0.5 and 0.6), but on some minor types, for example, percentage of (sporadic) plants, the correlation can be as low as 0.1. They did not observe a generally strong association between the greenspace metrics and the sleep characteristics of children in their cohort, but they did find that the associations differed by racial and socioeconomic subgroups. Larkin et al. (2021) also compared NDVI and street view data, but their target was the more general built environment rather than greenspace alone. Based on the literature we reviewed, we consider that the studies of direct comparison of the two currently most widely used methods of measuring greenspaces are still limited, and their findings are inconsistent.

Greenspace measurements based on aerial or satellite remote sensing data and those based on street view data take different approaches to quantify the greenness around a person/location. The former usually calculates the fraction of certain land use/land cover types or the intensity of NDVI value within one or more fixed‐radius buffer zones around the locations or routes of interest (e.g., Su et al., 2019). Analyses based on street view data typically calculate the Green View Index (GVI) by either examining the hue of pixels in the street view images (Larkin & Hystad, 2019) or recognizing objects based on semantic segmentation of the street view photos, and the latter is achieved through advanced deep learning and computer vision technologies (e.g., Helbich et al., 2019, 2021; Ki & Lee, 2021; Li, 2021; Xia et al., 2021), or both (Larkin et al., 2021). Since the goal is to emulate a person’s actual perception and also due to the photos being taken only from the street, the measures based on street view data are usually not calculated for multiple distances from the person’s location/trajectory.

The main theme of Verheij et al. (2008) we describe at the beginning of this paper is actually the discrepancy between the health situations in urban and rural areas. The authors consider that access to greenspace may significantly contribute to the neighborhood’s health status, at least as important as other factors in the classic theories about that discrepancy, such as migration, behavior, and exposure to pollution. However, the authors did not provide a detailed definition of greenspace in rural areas. Taylor and Hochuli (2017) found that the study of greenspace in the urban environment is dominant in this field, and the definition of greenspace is far from clear and consistent. Some definitions do not distinguish urban and rural (e.g., Lachowycz & Jones, 2013), and some explicitly take an urban perspective and treat the rural or semi‐rural areas entirely as greenspaces (e.g., Lange et al., 2008). Either way, they are similar in claiming or implying that the vegetated land in rural areas can be entirely perceived as greenspaces, and eventually only those immediately adjoining urban areas are meaningful greenspaces. Taylor and Hochuli (2017) raised the term *rural greenspace*, but they simply mention it as an implication derived from the term *urban greenspace*, without clarifying its meaning. They did not identify any studies explicitly about *rural greenspace*, either.

Meanwhile, Colley and Craig (2019) reviewed and discussed the distinction between wild versus tamed (or lightly vs. heavily managed) greenspace, for example, urban woodlands versus urban parks. Wolff et al. (2020) also state that previous studies have not systematically differentiated between greenspace types such as parks or forest areas. While their discussions are still largely within the urban context or on the rural‐urban interface, and from the perspective of how rural greenspaces can benefit the city population, if we consider that greenspace features viewability and accessibility, then this kind of distinction has a more profound implication in rural areas. This is because not all, and actually only a (small) portion of, vegetated land in rural areas have viewability and accessibility and thus can be considered as greenspace. On the other hand, the impact of greenspace on health may differ for people in urban and rural areas (Wheeler et al., 2015). Mitchell and Popham (2007) found no association between greenspace and health in higher‐income suburban and higher‐income rural areas, whereas, in lower‐income suburban areas, a higher proportion of greenspace was associated with worse health. They interpret the pattern as a result of the fact that besides quantity, the quality of greenspace is also significant in determining health benefits. Nishigaki et al. (2020) found that urban areas with higher tree density and rural areas with moderate amounts of grassland were associated with lower odds of depression. However, while there is a lack of understanding of how the rural greenspace is relevant to the rural population, and there is evidence that the impact of greenspace on human health is different in urban and rural areas, the literature is very limited.

As part of a birth cohort study, this study focuses on maternal exposure to residential greenspaces and its impact on birth outcomes. A number of previous studies have addressed this topic. A systematic review and meta‐analyses (Akaraci et al., 2020) covering 37 previous studies found that an increase in residential greenness was associated with higher birth weight (BW) and lower odds of small gestational age, whereas associations between greenspace and low birth weight (LBW) and preterm birth (PTB) were not. Most (27 out of 37) of the studies used NDVI within circular buffers around each mother’s residential address or even just the centroid of the areal unit of the address to measure the greenspace exposure, whereas the others used measures such as distance to major greenspaces in the neighborhood (e.g., parks), the proportion of greenspaces in the subjectively defined neighborhood, or the percentage of tree coverage and tree availability along the street. Those non‐NDVI‐based measures were based on land use/land cover data from remote sensing or other geographic information systems (GIS) sources. None of the reviewed studies used street view data. Another limitation of the studies reviewed is that most of them are about urban areas, with few covering the entire large administrative area without distinguishing between urban and rural areas. For example, Laurent et al. (2019) investigated the association between greenness and LBW using birth records from the entire state of California. This is also the case for some more recent studies not covered by the review given by Akaraci et al. (2020): they are either more interested in urban areas (e.g., Akaraci et al., 2021; Toda et al., 2020) or cover large areal units without distinguishing between urban and rural areas (e.g., Runkle et al., 2022; Sun et al., 2020; Vilcins et al., 2021). These more recent studies all measured greenspace based on NDVI or existing land use databases (likely also based on remote sensing data), and none incorporated street view data.

In the present study, we compared measures of greenspace based on the GSV data with those based on remotely sensed (RS) data. Specifically, we calculated the correlation between the greenspace measures based on different data and evaluated their associations with birth outcomes. Our study area consists of the states of New Hampshire and Vermont, USA, which are largely rural. We consider that such a comparison between the street view and satellite‐derived data sets is particularly meaningful in the study of the health impact of rural greenspace because, on the one hand, in rural areas, the pathway of greenspace is mainly about improving mental health and physical activities, rather than reducing physical hazards, such as noise and air pollution, hence, reflecting what people actually see and access is essential for the greenspace measurement; on the other hand, while the raw greenness is generally high in rural areas, not all vegetated land is visible and/or accessible in people’s daily lives. Unlike vertically sensed data, the street view data more directly represents viewable and accessible greenspaces. However, we are unaware of an empirical comparison of the two types of data for representing greenspace in rural areas in the literature.

## 2 Materials and Methods

### 2.1 Birth Outcome Data and Process

The participant data used in this study are from the New Hampshire Birth Cohort Study (NHBCS), which was designed to study the effects of environmental exposures on maternal and child health (Gilbert‐Diamond et al., 2011, 2016). More information about this cohort and research based on it has been well‐published (Chipman et al., 2024; Emond et al., 2018; Fleisch et al., 2020; Gilbert‐Diamond et al., 2011; Romano et al., 2019; Shi et al., 2015; Signes‐Pastor et al., 2019). Here are some descriptive statistics of the infant’s birth year: median = 2013 (25%–75% quartiles 2011–2015), mean = 2013, SD = 2.23, lower limit = 2009, and upper limit = 2018. We particularly provide these statistics, as it is important to compare this temporal information with the temporal properties of the environmental data used in this study. The participant data set used in this study contains geocodable addresses of 1,440 participants.

Among the 1,440 participants with geocoded addresses, we found useable street view data for 1,030 (71.5%). We included 1,030 participants in the following analysis.

### 2.2 Measures of Greenspace and Environmental Diversity Based on the Google Street View Data

**Figure 1:**
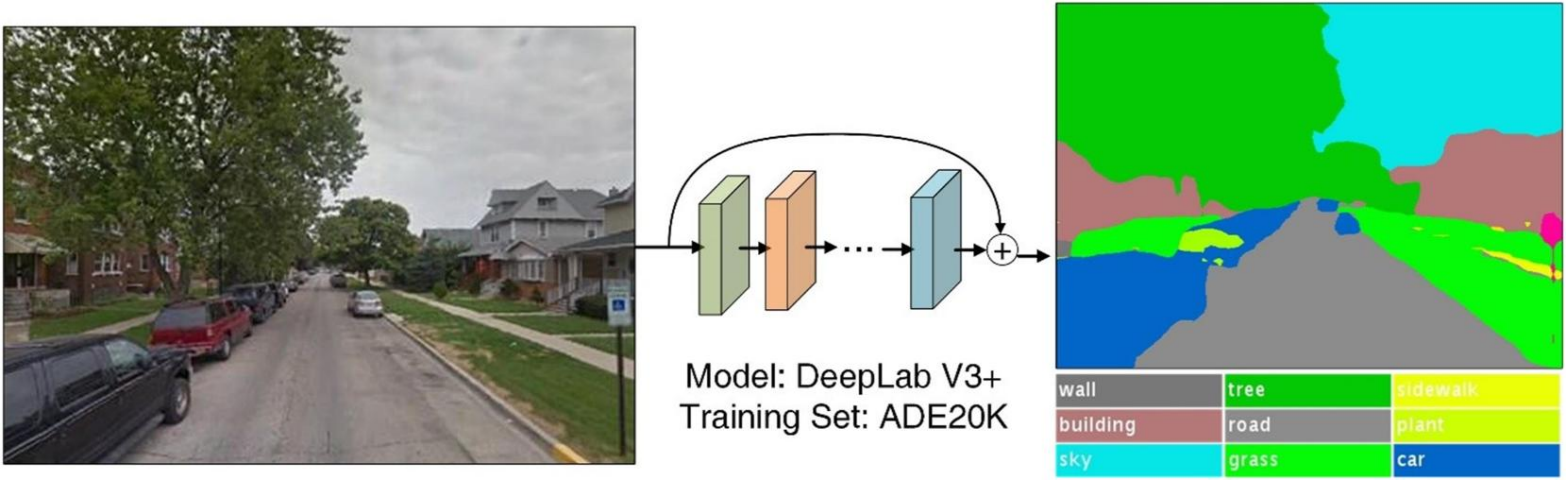
Image segmentation for building semantic visual element measures.

We derived greenspace measures from the results of object recognition applied to the GSV. The objects of different types in a street view image, for example, trees, grassland, roads, and houses, were recognized through image segmentation, as illustrated in Figure 1. Such image segmentation is typically achieved through a computer vision model. In this work, the particular computer vision model we used was DeepLab V3+ (Chen et al., 2018), trained on the ADE20K (Zhou et al., 2017) data set. The model can classify up to 150 object categories with over 82% pixel‐level accuracy.

In this study, we were able to find GSV images for 1,030 participants around their residential locations. We acquired all those GSV images through Google Cloud application programming interface (API) by requesting images within 100 m of a given location. Those images were mostly taken in 2010–2012. To represent a 360‐degree view of a location, we selected four GSV images for each location that were taken with the camera facing the four cardinal directions: north, east, south, and west. If one residential location has more than one GSV location around it, we acquired four images of each of those GSV locations.

**Figure 2:**
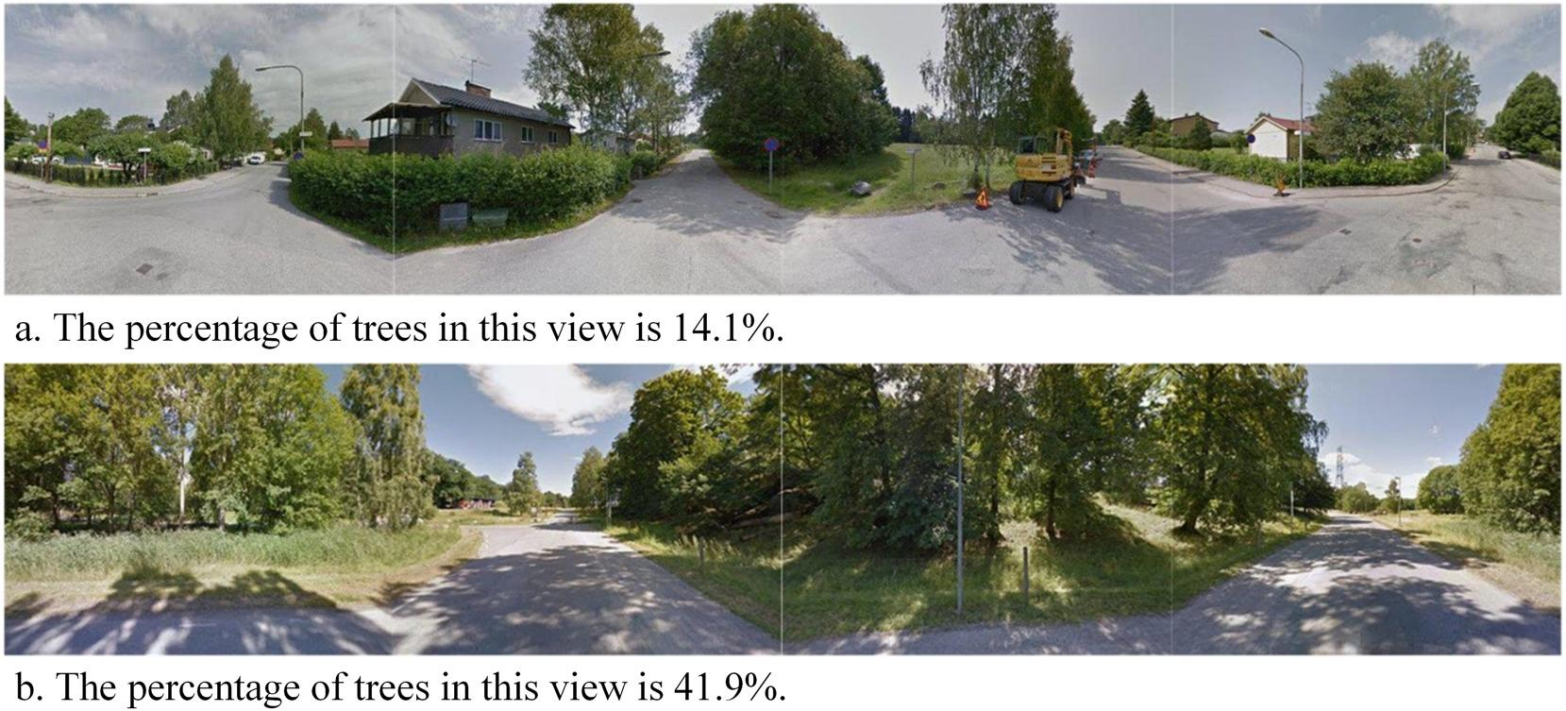
Illustrations of tree percentage in panoramas assembled from the Google Street View photos. Note: The sample locations are not from actual addresses from the present study. To protect the participants’ privacy, the images in this figure are not from our study area and are not part of the images we used in this study. The images are just for illustration.

To simulate a person’s view at a location, we assemble the four images at a GSV location into a panorama. We specified to have the DeepLab V3+ model to recognize 23 types of objects that might be relevant to this study, including trees, roads, sky, grass, ground, plants, agricultural fields, buildings, houses, sidewalks, cars, walls, fence, dirt road/track, water, rock/stone, water body, mountain, pole, floor, signboard, flower, and people. All the other types were attributed to “others”; therefore, the total number of types considered in the following analysis was 24. We then calculated the percentage of each type in the assembled panorama, as illustrated in Figure 2. In the case that one residential location has more than one GSV location around it, we used the average of the percentages of an object type based on all sets of images as the final percentage of that type at that residential location.

Finally, we calculated the measure of visible and accessible greenspace at a location as the sum of percentages of five types of objects:

(1) Greenspace=tree+grass+plant+agriculturalfield+flower

Besides the measure of visible and accessible greenspace, because the DeepLab V3+ model was so powerful in recognizing different categories of objects in the image, we decided to also calculate measures of environmental diversity at a residential location based on the objects recognized by the model. This diversity analysis was inspired by the work of Hanski et al. (2012), which investigated the association of environmental biodiversity with allergies and other chronic inflammatory diseases among urban populations. They found that environmental biodiversity around the study subjects’ residences influenced the composition of the bacterial classes on their skin. For a large number of residence sites, it is difficult to directly measure the environmental biodiversity around each, that is, it is difficult to directly count the number of different types of biological species and measure the quantity of each. To overcome this difficulty, in this study, we measured the diversity of environmental features (e.g., different types of vegetation and land cover), so‐called “environmental diversity”, as a substitute for environmental biodiversity. This substitution is based on the assumption that within a limited geographic area that features relatively consistent environmental conditions, such as climate, vegetation, and land use pattern, the environmental diversity can be a good representation of the actual environmental biodiversity. In this study, we calculated Shannon’s Index (entropy) of different object types as a measure of environmental diversity:

(2) $$H=-\sum_{i=1}^{s}p_{i}\;\ln\;p_{i}$$

where *H* is Shannon’s Index; *s* is the total number of object types recognized from the GSV images (24 in this study); *p*_*i*_ is the percentage of pixels labeled as object *i* in the image.

### 2.3 Measures of Greenspace and Environmental Diversity Based on Remotely Sensed Data

To compare with the GSV‐based greenspace and environmental diversity measures, we derived corresponding measures from RS data, referred to as *RS‐based measures* herein. To calculate RS‐based measures, we constructed circular buffer zones with radii of 100, 250, 500, 1,000, 2,000, and 3,000 m around all subjects’ locations. We then calculated the RS‐based measures within each buffer zone. We chose 100 m as the radius for the first buffer zone in the series, assuming it defines the neighborhood within which the greenness is directly viewable and accessible to the resident at the location, and thus, its greenspace measures are most comparable with the GSV‐based ones. The choice of 100 m also takes into account the resolution of the RS data (30 m). The rest of the radii form a sequence to reveal the distance decay of the comparability between the measures based on the two types of data. Table 1 summarizes the names, creation, and data sources of the RS‐based measures. The selection and derivation of these RS‐based measures follow Chipman et al. (2024).

**Table 1:** Greenspace and Environmental Diversity Measures Based on Remotely Sensed Data

Name	Description	Data source	Value range
LC	Fraction of a buffer area around each participant’s location that is composed of open‐space land cover types, including forest, grassland, etc. Basically, it is all classes except the developed classes	US National Land Cover Database 2011 (NLCD archive, https://www.mrlc.gov/data)	0–1
IS	Fraction of a buffer area around each participant’s location that is impervious surfaces (roads, rooftops, etc.) This is inversely related to greenspace	NLCD archive	0–1
TC	Average tree canopy cover fraction within the buffer area around each participant’s location	NLCD archive	0–1
VI	Average of the NDVI value within the buffer area	Landsat‐8 multispectral images from Worldwide Reference System‐2 (WRS‐2)	−1–1
Shannon’s Index (Diversity)	Shannon’s Index based on the fraction of different land cover types within a buffer zone of a residential location. In this study, we only calculated this Index for the 100‐m neighborhood of each location, because it is the most comparable with the diversity measure based on the GSV data	US National Land Cover Database 2011 (NLCD archive, https://www.mrlc.gov/data)	0—ln(s), where *s* is the number of types

### 2.4 Assessment of Consistency Between the Greenspace and Diversity Measures Based on Different Data

We calculated Pearson’s correlation coefficient between the GSV‐based greenspace and diversity and the RS‐based measures described in 2.3. This comparison intends to quantify the consistency and distinction between the human eye’s view and the bird eye’s view. Different ground objects and their patterns may be visible or invisible in those two views. Thus, to have a better understanding of what is specifically contributing to the consistency and distinction between the two types of measurements, or in other words, to detect if specific objects recognized from the GSV data are particularly correlated to different RS‐based greenspace measures, besides the *overall greenness* calculated using Equation 1, we also calculated the correlation coefficient between certain combinations of green objects and the RS‐based greenspace measures.

### 2.5 Detection of Associations Between Birth Outcomes and Greenspace Measures

Multivariate regression was used to model the relationship between each greenspace metric and each of the four dependent variables, controlling for the confounding effects of a suite of other demographic and pregnancy‐related variables. Dependent variables included length, weight, head circumference z‐scores, and gestational age in weeks. Covariates included maternal age, delivery type (vaginal or c‐section), parity, maternal educational level (high school or less, junior college graduate or some college, college graduate, any postgraduate schooling), maternal insurance (private or public/other), self‐reported exercise during pregnancy (yes/no), birth season, smoking history (yes/no), child’s sex, pre‐pregnancy body mass index, maternal race/ethnicity, and maternal prenatal total urinary arsenic.

The modeling was performed in R using the Robust Regression method (Hampel et al., 2011; Huber, 2004; Marazzi, 1993) as implemented in the Robust Fitting of Linear Models (rlm) algorithm, owing to its robust handling of outliers, with 10‐fold cross‐validation. Before regression modeling, 10 replications of the Multivariate Imputation by Chained Equations algorithm were used to estimate any missing values of controls/covariates (Van Buuren & Groothuis‐Oudshoorn, 2011). More elaborated description and discussion of the selection of the covariates, statistical analysis, and test of the statistical significance of the results can be found in another recently published paper, which presents a study based on the same cohort but focuses on the greenspace measured based on the RS data (Chipman et al., 2024).

For each measurement of birth outcome, we ran three regression analyses, as described in Table 2. In the regression analyses, each birth outcome was the dependent variable, with independent variables including the measures of greenspace and/or environmental diversity, calculated based on different data (GSV or RS), along with the maternal and infant factors. For the RS‐based measures, we only ran the regression analyses for the buffer of 0–100 m, because this smallest buffer is the one that is most comparable with the results from the GSV‐based measures.

**Table 2:** The Regression Analysis Design of Birth Outcome Measurements Against Greenness and Environmental Diversity Measured Based on the GSV and RS Data, Considering Maternal and Infant Factors

Dependent	Independent	Measurements	Model 1	Model 2	Model 3
• z‐score of height at birth	*Greenness*	• See Equation 1 for the GSV‐based measures	X		X
		• See Table 1 for the RS‐based measures			
• z‐score of height‐based weight at birth	*Environmental diversity (Shannon's Index)*	• See Equation 2 for the GSV‐based measure		X	X
		• See Table 1 for the RS‐based measure			
• z‐score of head circumference at birth	Maternal age	The age	X	X	X
	Delivery type	• Vaginal	X	X	X
• Gestational age		• C‐section			
	Parity	0, 1, 2, 3+	X	X	X
• Classified as small by gestational age	Maternal education level	• High school or less	X	X	X
		• Junior college graduate or some college or technical school			
		• College graduate			
		• Postgraduate schooling			
	Maternal Insurance	• Private	X	X	X
		• Public or other			
	Maternal exercise during pregnancy	• No	X	X	X
		• Yes			
	Birth season	• Spring	X	X	X
		• Summer			
		• Fall			
		• Winter			
	Smoking status	• Never	X	X	X
		• Past or current			
	Baby male	• No	X	X	X
		• Yes			
	Pre‐pregnancy body mass index	Log transformed	X	X	X
	Maternal race/ethnicity	• White and not Hispanic/Latino	X	X	X
		• All other			
	Urinary arsenic (log‐transformed)	Log transformed	X	X	X

## 3 Results

### 3.1 Correlation Between the Measures of Greenspace Based on the Two Types of Data

Tables 3, 4, 5, 6, 7 show Pearson’s correlation coefficients between the GSV‐based measures of greenspace and RS‐based measures. Findings from those results include:

(1) In our rural study area, GSV‐based measures had low to moderate correlations with the RS‐based measures: all *r* values are within the range [−0.361, 0.433].
(2) None of the five GSV‐based greenness measures had a particularly strong or weak correlation with the RS‐based measures. On the other hand, none of the four RS‐based measures stood out to be particularly more strongly or weakly correlated with the GSV‐based measures.
(3) The GSV‐based measures are generally more correlated with the RS‐based measures for a small buffer around the residential location. As the buffer widened, the correlation decreased. The only exception to that distance‐decay pattern is the correlation about the tree canopy. The GSV‐based measures had the strongest correlation with the tree canopy in the 0–250 m neighborhood. When only trees in the Google View data are considered, *r* = 0.433 for the 0–250 m neighborhood, the highest among all *r* values.
(4) The correlations about the open‐space type are almost exactly opposite to their counterparts about the impervious surface type, indicating these two types of land cover are almost complementary in a typical rural residential neighborhood.
(5) The GSV‐based measure of environmental diversity was very weakly correlated with the RS‐based measure of diversity (Table 7).

**Table 3:** Pearson’s Correlation (*r*) Between Greenness Measures Based on the Google Street View Data and the Measures Based on Open‐Space Land Cover (LC in Table 1; *N* = 1,030)

	Buffer for the measure based on the opens‐space land cover					
	0–100 m	0–250 m	0–500 m	0–1,000 m	0–2,000 m	0–3,000 m
Tree + Grass + Plant + Agricultural Field + Flower (Overall greenness)	0.343	0.256	0.197	0.176	0.140	0.110
Tree + Grass + Plant + Agricultural Field	0.343	0.256	0.197	0.176	0.140	0.110
Tree + Grass + Plant	0.333	0.243	0.184	0.164	0.130	0.101
Tree + Grass	0.327	0.238	0.177	0.156	0.127	0.102
Tree	0.370	0.284	0.212	0.187	0.158	0.130

**Table 4:** Pearson’s Correlation (*r*) Between Greenness Measures Based on the Google Street View Data and the Measures Based on Impervious Surfaces (IS in Table 1; *N* = 1,030)

	Buffer for the measure based on the impervious surface					
	0–100 m	0–250 m	0–500 m	0–1,000 m	0‐2,000 m	0–3,000 m
Tree + Grass + Plant + Agricultural Field + Flower (Overall greenness)	−0.341	−0.244	−0.185	−0.167	−0.135	−0.108
Tree + Grass + Plant + Agricultural Field	−0.341	−0.244	−0.185	−0.167	−0.136	−0.108
Tree + Grass + Plant	−0.330	−0.231	−0.173	−0.155	−0.126	−0.099
Tree + Grass	−0.324	−0.226	−0.166	−0.148	−0.123	−0.100
Tree	−0.361	−0.263	−0.196	−0.176	−0.151	−0.126

**Table 5:** Correlation (*r*) Between Greenness Measures Based on the Google Street View Data and the Measures Based on Tree Canopy Cover (TC in Table 1; *N* = 1,030)

	Buffer for the measure based on the tree canopy cover					
	0–100 m	0–250 m	0–500 m	0–1,000 m	0–2,000 m	0–3,000 m
Tree + Grass + Plant + Agricultural Field + Flower (Overall greenness)	0.110	0.338	0.244	0.186	0.128	0.094
Tree + Grass + Plant + Agricultural Field	0.109	0.338	0.244	0.186	0.128	0.094
Tree + Grass + Plant	0.096	0.343	0.248	0.183	0.125	0.091
Tree + Grass	0.096	0.344	0.242	0.168	0.111	0.083
Tree	0.055	0.433	0.328	0.248	0.186	0.152

**Table 6:** Correlation (*r*) Between Greenness Measures Based on the Google Street View Data and the Measures Based on NDVI (VI in Table 1; *N* = 1,030)

	Buffer for the measure based on NDVI					
	0–100 m	0–250 m	0–500 m	0–1,000 m	0–2,000 m	0–3,000 m
Tree + Grass + Plant + Agricultural Field + Flower (Overall greenness)	0.370	0.262	0.221	0.200	0.166	0.124
Tree + Grass + Plant + Agricultural Field	0.371	0.263	0.222	0.200	0.166	0.124
Tree + Grass + Plant	0.361	0.251	0.212	0.191	0.158	0.117
Tree + Grass	0.352	0.244	0.201	0.176	0.150	0.112
Tree	0.373	0.268	0.224	0.201	0.169	0.141

**Table 7:** Correlation (*r*) Between Environmental Diversity Measured With the Google Street View Data and the Diversity Measured With the Land Cover Data (*N* = 1,030)

	Buffer for the diversity measured with the land cover data					
	0–100 m	0–250 m	0–500 m	0–1,000 m	0–2,000 m	0–3,000 m
Diversity based on GSV	0.155	0.165	0.160	0.101	0.071	0.069

### 3.2 Associations of Birth Outcomes With Greenness and Environmental Diversity Measures

Table 8 compares the associations of birth outcomes with the greenness measured based on the GSV data and measured based on the RS data (Model 1 in Table 2). In our results, none of the birth outcomes is associated with the GSV‐based greenness measure at the level of *α* = 0.05. The RS‐based VI is significantly associated with the birth height (negatively) and the height‐based weight (positively).

**Table 8:** Associations Between Birth Outcomes and the Greenspace Measures Based on the Google Street View Data (Model 1 in Table 2; Refer to Table 1 for the Meaning of RS‐Based Measures; the RS Measures Are Only for the Buffer of 1–100 m; *N* = 1,030)

Greenspace measures	Statistics	Birth outcomes					
		Height	Weight	Height‐based weight	Head circumference	Gestational age	Classified as small by gestational age
GSV	Coeff.	0.176	0.221	−0.051	0.149	0.515	0.209
	*P*‐Value	0.605	0.361	0.886	0.649	0.118	0.842
LC	Coeff.	−0.178	0.034	0.235	−0.043	0.040	0.210
	*P*‐Value	0.402	0.822	0.288	0.833	0.844	0.741
IS	Coeff.	0.270	−0.279	−0.716	−0.256	−0.250	−0.301
	*P*‐Value	0.518	0.352	0.101	0.517	0.528	0.807
TC	Coeff.	0.272	0.471	0.485	0.702	0.459	−0.399
	*P*‐Value	0.493	0.096	0.239	0.063	0.229	0.738
VI	Coeff.	−3.410	−0.971	2.782	−1.084	−0.998	4.538
	*P*‐Value	0.006^a^	0.214	0.032^b^	0.349	0.394	0.220

^a^ Statistically significant at the 0.01 level.

^b^ Statistically significant at the 0.05 level.

Table 9 shows results about environmental diversity (Model 2 in Table 2). The gestational age was inversely associated with environmental diversity measured with the GSV data. No other clear associations were observed.

**Table 9:** Associations Between Birth Outcomes and Environmental Diversity Calculated Based on Various Measures (Model 2 in Table 2; the Diversity Measured With the Land Cover Data Is Only for the Buffer of 1–100 m; *N* = 1,030)

Diversity	Statistics	Birth outcomes					
		Height	Weight	Hight‐based weight	Head circumference	Gestational age	Classified as small by gestational age
GSV	Coeff.	−0.133	−0.251	−0.282	−0.010	−0.530	−0.446
	*P*‐Value	0.586	0.154	0.269	0.968	0.039^a^	0.575
Land cover	Coeff.	0.067	0.034	−0.090	−0.077	0.045	−0.252
	*P*‐Value	0.623	0.727	0.532	0.556	0.732	0.523

^a^ Significant at 0.05 level.

Table 10 shows the regression analysis results in which both greenness and environmental diversity were included as independent variables (Model 3 in Table 2). The results are similar to the results from Models 1 and 2. No association between the infant outcomes and the GSV‐based measures was observed. Again, the RS‐based vegetation index (VI) is significantly associated with the birth height (negatively) and the height‐based weight (positively).

**Table 10:** Associations Between Birth Outcomes and Both Landscape Diversity and Greenness, Calculated Based on the Two Types of Data (Model 3 in Table 2; *N* = 1,030; Refer to Table 1 for the Meaning of RS‐Based Measures; the RS Measures Are Only for the Buffer of 1–100 m)

Measures		Statistics	Birth outcomes					
			Height	Weight	Hight‐based weight	Head circumference	Gestational age	Classified as small by gestational age
GSV	Greenness	Coeff.	0.134	0.139	−0.174	0.174	0.280	−0.064
		*P*‐Value	0.705	0.585	0.638	0.633	0.443	0.956
	Diversity	Coeff.	−0.106	−0.225	−0.318	0.047	−0.439	−0.468
		*P*‐Value	0.679	0.220	0.234	0.867	0.122	0.598
RS	LC	Coeff.	−0.163	0.050	0.214	−0.094	0.068	0.097
		*P*‐Value	0.458	0.750	0.350	0.660	0.752	0.882
	Diversity	Coeff.	0.041	0.041	−0.057	−0.097	0.058	−0.233
		*P*‐Value	0.772	0.682	0.700	0.480	0.674	0.573
	IS	Coeff.	0.234	−0.327	−0.690	−0.198	−0.310	−0.102
		*P*‐Value	0.589	0.292	0.128	0.629	0.451	0.935
	Diversity	Coeff.	0.046	0.061	−0.030	−0.058	0.072	−0.243
		*P*‐Value	0.743	0.350	0.840	0.672	0.596	0.552
	TC	Coeff.	0.283	0.476	0.465	0.684	0.478	−0.491
		*P*‐Value	0.478	0.094	0.262	0.072	0.213	0.681
	Diversity	Coeff.	0.072	0.038	−0.074	−0.050	0.063	−0.272
		*P*‐Value	0.599	0.698	0.604	0.706	0.634	0.495
	VI	Coeff.	−3.407	−0.964	2.777	−1.285	−0.954	4.216
		*P*‐Value	0.007^a^	0.219	0.036^b^	0.280	0.425	0.266
	Diversity	Coeff.	0.002	0.028	−0.003	−0.106	0.023	−0.147
		*P*‐Value	0.990	0.777	0.984	0.427	0.866	0.717

^a^ Statistically significant at the 0.01 level.

^b^ Statistically significant at the 0.05 level.

## 4 Discussion and Conclusions

We applied street view data to evaluate associations between birth outcomes and greenspace in a rural region of the USA. Our original hypothesis was that in a rural area like that in northern New England, where densely vegetated land is dominant, the eye‐level horizontal view of greenness measured based on the street view data could be quite different from the vertical view of greenness measured based on satellite imageries, and therefore more closely associated with birth outcomes than satellite views. As expected, the correlations between the two types of greenness measures (GSV‐based and RS‐based) were weak to moderate and decreased as the buffer around the residence expanded (from 100 to 3,000 m), indicating that the greenness at an eye‐level view reflects the immediate surroundings. Even for the smallest buffer (100 m), the association between the two types of measures was generally low (|*r*| < 0.5).

None of the GSV‐based measures of greenness were associated with any of the seven birth outcomes considered in this study. Among all RS‐based greenness, only the VI has significant associations with birth height (negatively) and height‐based weight (positively). This finding indicates that the greenspace in rural areas may influence birth outcomes differently than in urban areas.

Chipman et al. (2024) discussed two possible reasons why the greenspace appears to function differently in rural areas than in urban areas in terms of influencing birth outcomes, which we concur with and want to elaborate further on. First, they argue that the beneficial influence of greenspace on the birth outcome is not likely to increase linearly along with the increase of greenspace. Instead, the marginal effect may decrease as the greenspace increases and the influence finally reaches a plateau. In rural areas, it is likely that the benefit of being simply green may have been already saturated at most locations. Therefore, the birth outcomes would not be sensitive to the variation of greenness across those locations. Second, to the people in rural areas, the equation “natural = healthy” is questionable. So far, most studies of greenspace focus on urban areas, and many have found a positive influence of greenspace on human health, which is not surprising, because the studies basically reveal a longing and need for nature of those people stuck in the artificial built environment. The people in rural areas, however, may not have a similar psychological longing for the natural environment, as they are constantly submerged by such an environment. On the other hand, the wilderness may hamper the rural people from taking advantage of certain healthy factors, such as clean energy and good access to healthcare services.

Besides the direct measures of greenness, we also calculated the environmental diversity, using both the street view data and the land cover data. We hypothesized that environmental diversity was a healthy factor. However, the findings from this particular study do not support this hypothesis. In fact, it turned out that the environmental diversity measured with our data and methods was by‐large negatively associated with the birth outcomes, although only the association about gestational age was statistically significant. This may indicate that environmental diversity by itself is not necessarily a positive environmental factor for birth outcomes, especially in rural areas. Nevertheless, the health implication of environmental diversity is a topic and direction that warrants much more exploration. Some important issues awaiting further investigation and clarification include the actual meaning of landscape‐level environmental diversity and optimal data, methods and scales for measuring this kind of diversity, the representativeness of environmental diversity for biodiversity, and the pathway of the health implications of environmental diversity. We hypothesize that the findings about these issues will be context‐specific and vary across different regions, health outcomes, and research questions. Our study is an initial effort of such an exploration.

A major limitation of this study is that we could only find useable GSV images for about 70% of the cohort participant’s residential locations. Those residences may be located in places more accessible to GSV vehicles, for example, major roads, and thus may not be well representative of all women who had given birth in our study area.

Another limitation is that we did not consider the seasonality and yearly variability of the GSV data. A mother’s interaction with greenspace at a given time in her pregnancy would depend on the seasonality of the pregnancy. In other words, the difference between high versus low amounts of greenspace at a given location might matter more in spring/summer than in winter, so pregnancies during spring/summer might be more influenced by greenspace.

An additional limitation of this study is that, while we are aware that it is particularly important in rural areas to consider both visibility and accessibility of greenspace when assessing its health impact, this study was only able to work on the greenspace directly visible from the mother’s residence. The accessibility of greenspace, especially in rural areas, is challenging to measure. For example, to have a good knowledge of accessible greenspace, we need detailed information about vegetation types, public facilities (e.g., parks and hiking trails), and residents’ physical activities and daily movements. Incorporating those greenspaces that residents would access but not directly visible from their residences, for example, greenness along hiking trails, is on our research agenda.

Still another limitation is that in this study, we only measured greenness but did not try to recognize facilities with intensive design and management, for example, parks and gardens. In other words, we were only considering the potential psychological influence of greenspace but did not address its second important function: facilitating outdoor physical activities and social connections.

Finally, regarding access to greenspace, the situation of rural residents might be more complicated than that of urban residents. For example, residents of a small town may have a situation more like urban residents, that is, the greenspace they access is more likely to be managed (for example, public parks), whereas residents in more remote locations may be surrounded by less managed greenness, and there are a series of intermediate states between the two. In this study, however, we lumped all samples (situations) into the same model.

To address the above limitations and go beyond, topics we intend to include in future research agenda include: (a) integrating the street view data, RS data, and existing GIS data in measuring greenspace, as proposed and explored by Barbierato et al. (2020)—our motivation for doing this is to using the RS data to distinguish the general environment (e.g., residents of towns or in remote areas), and use the GSV data to recognize more local and detailed viewable and accessible greenness; (b) measuring greenspace based on a person’s footpath view along daily mobility trajectories, rather than just around the residential location; and (c) incorporating blue space (water bodies) and white space (snow), which also could influence human health (Akaraci et al., 2020).

## Conflict of Interest

The authors declare no conflicts of interest relevant to this study.

## Data Availability

The birth outcome data used in this research are considered protected health information and are not publicly available. Users wishing to negotiate access to the data under the terms of the Dartmouth CPHS and the Dartmouth Institutional Review Board should contact the principal investigator, Dr Margaret Karagas. Geospatial data (land cover, impervious surface area, and tree canopy cover) are available from Multi‐Resolution Land Characteristics Consortium (https://www.mrlc.gov/). Google Street View images around the participants’ residential locations can be acquired from Google Cloud API by requesting images within 100 m of a given location. The analyses were conducted in R statistical software and the associated packages are openly available from Kuhn (2008) and Wickham et al. (2019).
